# Resurrection of DNA Function *In Vivo* from an Extinct Genome

**DOI:** 10.1371/journal.pone.0002240

**Published:** 2008-05-21

**Authors:** Andrew J. Pask, Richard R. Behringer, Marilyn B. Renfree

**Affiliations:** 1 Department of Molecular Genetics, University of Texas M. D. Anderson Cancer Center, Houston, Texas, United States of America; 2 Department of Zoology, University of Melbourne, Melbourne, Victoria, Australia; Lund University, Sweden

## Abstract

There is a burgeoning repository of information available from ancient DNA that can be used to understand how genomes have evolved and to determine the genetic features that defined a particular species. To assess the functional consequences of changes to a genome, a variety of methods are needed to examine extinct DNA function. We isolated a transcriptional enhancer element from the genome of an extinct marsupial, the Tasmanian tiger (*Thylacinus cynocephalus* or thylacine), obtained from 100 year-old ethanol-fixed tissues from museum collections. We then examined the function of the enhancer *in vivo*. Using a transgenic approach, it was possible to resurrect DNA function in transgenic mice. The results demonstrate that the thylacine *Col2A1* enhancer directed chondrocyte-specific expression in this extinct mammalian species in the same way as its orthologue does in mice. While other studies have examined extinct coding DNA function *in vitro,* this is the first example of the restoration of extinct non-coding DNA and examination of its function *in vivo*. Our method using transgenesis can be used to explore the function of regulatory and protein-coding sequences obtained from any extinct species in an *in vivo* model system, providing important insights into gene evolution and diversity.

## Introduction

Extant species represent less than 1% of the genetic diversity that has existed in the animal kingdom [1]. Extinction rates are increasing at an alarming rate, especially of mammals [2], [3]. Many efforts, such as that of the Frozen Zoo (San Diego Zoological Society Conservation and Research for Endangered Species) are working to cryo-archive cell and tissue resources from a diverse range of threatened species, to protect their genetic information. However, for those species that have already become extinct, access to their genetic biodiversity may not be completely lost.

**Figure 1:**
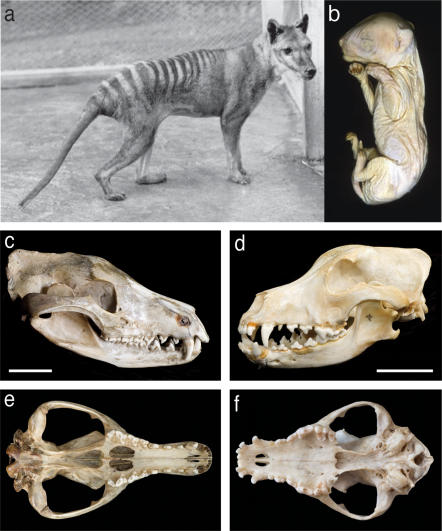
The thylacine, *Thylacinus cynocephalus.* (a) Young male thylacine in Hobart Zoo in 1928, photograph (Q4437). (b) One of the preserved pouch young specimens (head length 34 mm) from which DNA was extracted, from the Museum Victoria collection. (c-f) The skull of the thylacine (c,e) compared with that of the domestic dog *Canis canis* (d,f). The morphology of the head shows remarkable convergent evolution. However, there are some differences: in marsupials, the lacrymal extends outside the orbit and the angle of the dentary is medially inflected (c). The thylacine palatine has the vacuities characteristic of marsupial skulls (e). The teeth also show striking convergent evolution but the muzzle of the thylacine is quite narrow compared to that of the dog (e,f). Scale bar = 5cm.

The Tasmanian tiger or thylacine (*Thylacinus cynocephalus*) was a large, carnivorous Australian marsupial. Often described as the most striking example of convergent evolution in the mammalian lineage, the marsupial thylacine was morphologically almost indistinguishable from the eutherian canids, apart from the presence of a pouch where its young developed (Figure 1) [4]. Thylacines were hunted to extinction in the wild in the early 1900s and the last known animal died in captivity in the Hobart Zoo in 1936 [4]. Fortunately, some thylacine pouch young and adult tissues were preserved in alcohol in several museum collections around the world (Figure 1b).

With improved techniques for the isolation of ancient DNA, it is now possible to access the genomes of extinct species [5]. There have been several recent papers published examining the genomes from a diverse range of extinct species from plants and bacteria to mammoths and Neanderthals [2], [5]–[7]. While most studies to date have examined the mitochondrial DNA of these species for phylogenetic purposes, the focus is now shifting to the analysis of genomic DNA. Recent advances in sequencing technologies, such as direct high-throughput parallel pyrosequencing, are expected to expand the ancient DNA data bank exponentially over the coming years [5]. Most studies have used the sequence data to examine divergence times and population structure of extinct species. However, more recently this information is being used to examine how the function of these genes may have evolved. The function of the *Melanocortin 1 Receptor* (*MC1R*) gene has recently been investigated from the extinct mammoth and Neanderthal [1], [7]. Isolated *MC1R* sequences from ancient DNA samples were cloned and transfected into cell lines to examine the function of the receptor in activation assays *in vitro.* The results of these experiments suggest that variation in skin and hair pigmentation may have occurred within these species [1], [7] and describes a technical platform for examining extinct protein function *in vitro*.

While many adaptive changes throughout evolution have been ascribed to changes within the proteins themselves [8], [9], the high conservation of protein coding regions between mammalian genomes suggests that changes in the open reading frames are unlikely to be the primary cause of the vast differences observed in both form and function [10]–[12]. With the recent release of the ENCODE pilot study [13] it is clear that the majority of the non-coding mammalian genome is transcribed. It appears that rather than changes in the genes themselves, it is subtle differences in their non-coding regulatory elements that control their spatial, temporal and quantitative expression that underpins the variation in the animal kingdom [10]–[12]. Therefore, the non-coding regions of extinct genomes are likely to hold the most important genetic information that defined a species. We have therefore developed a technique for examining the function of non-coding regions from extinct genomes *in vivo*. To gain a greater insight into the function of these elements we isolated a regulatory sequence from the genome of the extinct thylacine using transgenesis and examined its function in mice.

## Results

### Extinct DNA Isolation and Fragment Characterisation

**Figure 2:**
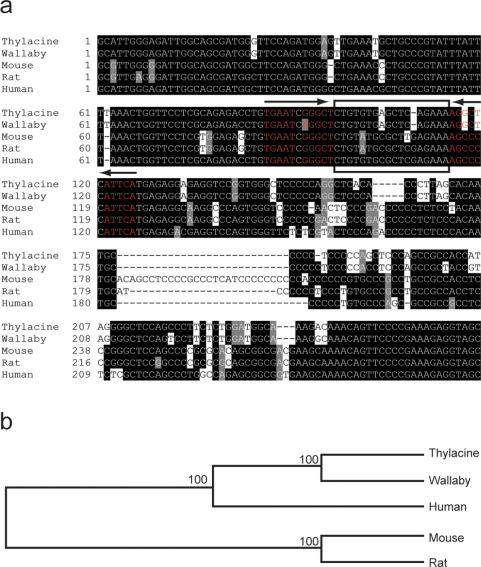
Analyses of the thylacine *Col2a1* enhancer element. (a) Sequence alignment of the thylacine PCR cloned *Col2a1* enhancer. Identical alignment between species is shown by black boxes. The thylacine sequence was most similar to, but distinct from that of another marsupial, the tammar wallaby, *Macropus eugenii*. The black box outlines the minimal 18-bp element. Inverted repeats (arrows) surrounding the minimal element are shown with complementary nucleotides in red. The outer repeat is highly conserved between all mammals, but the inner repeat differs by 2 nucleotides in the thylacine. (b) Phylogenetic analysis of the thylacine *Col2a1* enhancer. The thylacine sequence groups with the tammar wallaby, both of which are more similar to the human sequence than to mouse or rat. Numbers indicate bootstrap values based on 100 replicates.

To resurrect the function of non-coding DNA from an extinct mammal, we isolated genomic DNA using the technique described by Pääbo [14]. DNA was obtained from four 100-year-old specimens: three alcohol-fixed pouch young (one shown in Figure 1b) and one dried adult pelt, obtained from Museum Victoria (Melbourne, Victoria, Australia). The isolated DNA was fragmented as expected [6], and ranged from 300–500 bp. We obtained 200 ng to 1 μg from each sample. We chose to isolate the well-characterised transcriptional enhancer element of the proα1(II) collagen (*Col2a1*) gene [15]–[17]. This element was chosen because it is relatively conserved among mammals and directs chondrocyte-specific expression in the mouse [15], [17]. Primers were designed to span the core enhancer from regions conserved across all described mammalian species using a Clustal-W alignment. Primers were engineered with *Spe*1 (forward primer) and *Xba*1 (reverse primer) restriction sites to allow for multimerisation of the resulting product (see Materials and Methods). We performed PCR amplification from each DNA sample independently to reduce the risk of cross contamination, and to ensure the sequence was identical across all individuals, confirming it was thylacine in origin. This approach was also used to ensure sequence obtained was not harbouring any genetic changes caused by spontaneous hydrolysis or oxidation of the preserved DNA [6]. The resulting 264-bp PCR product from each sample, encoding the enhancer was independently subcloned and sequenced. The sequence obtained from each of the four independent thylacine samples was identical and a BLAST search confirmed it was different from all those available in the database and from the most likely sources of contamination in the lab (human and mouse) (Figure 2a). Phylogenetic analyses of the thylacine sequence with that of eutherian and marsupial species confirmed its identity as the thylacine *Col2a1* enhancer orthologue (Figure 2b), as it was most closely related to the tammar wallaby (*Macropus eugenii*, an extant marsupial species) than to human, mouse or rat.

### Transgenesis and Functional Analyses

**Figure 3:**
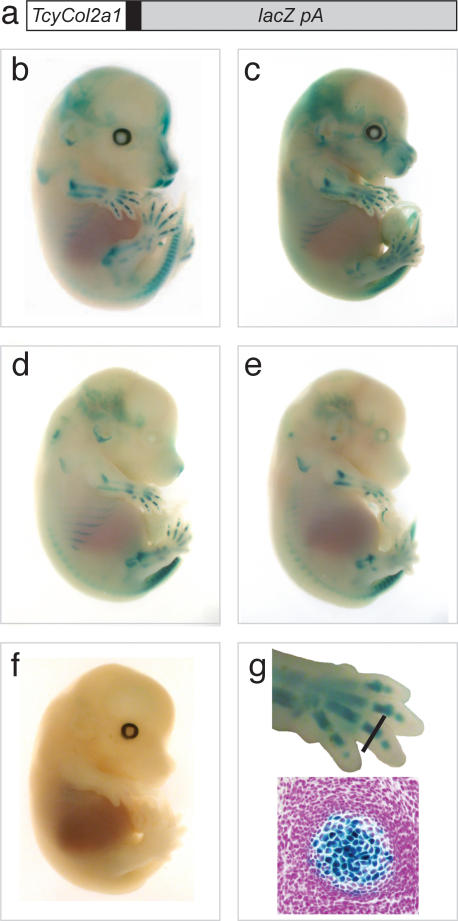
From extinction to gene expression. Functional analysis of the thylacine non-coding DNA fragment. (a) Diagram of transgene construct. 4 copies of a 264-bp fragment containing the Thylacine *Col2a1* enhancer (*TcyCol2a1*) region was ligated to the human b-globin minimal promoter (black box) and ligated to *lacZpA*. (b–e) X-gal stained 14.5 dpc *TcyCol2a1-lacZpA* transgenic mouse embryo showing varying levels of reporter gene expression within the developing cartilage (blue). (f) Non-transgenic littermate, negative control fetus. (g) Top panel; Magnified image of forelimb from fetus in (b) black line indicates the plane of section shown in (g) bottom panel. Bottom panel; Histological section of transgenic forelimb digit, showing *lacZ*-expressing chondrogenic tissue (blue) counterstained with eosin (pink).

The strength of the *Col2a1* regulatory element can be enhanced by using multiple copies of this region upstream of a reporter gene [16]. Four copies of the thylacine sequence were multimerised and ligated to the human β-globin basal promoter fused to *lacZ* and followed by a polyadenylation signal. The 4.75 kb *TcyCol2a1-lacZ-pA* construct (Figure 3a) was purified from the vector and microinjected into the pronuclei of mouse zygotes [18]. *LacZ* expression was examined in founder mice at 14.5 days *post coitum*, by staining with X-gal [18] (5-bromo-4-chloro-3-indolyl-b-D-galactopyranoside) (Figure 3b–e).

Whole mount staining was observed in all 4 founder fetuses created in a pattern indicative of developing cartilage (Figure 3b–e). This pattern was identical to that of the endogenous mouse *Col2a1* gene and mouse enhancer transgenes [15]–[17]. Histological cross sections of the developing limb confirmed that *lacZ* expression, directed by the thylacine enhancer element, was restricted to the developing chondrocytes (Figure 3g). Non-transgenic littermates were used as negative controls (Figure 3f) and showed no staining.

## Discussion

A non-coding DNA fragment from an extinct mammal was able to drive expression of a reporter gene construct in a developing fetus. While the intensity of the reporter gene expression seen in the transgenic fetuses varied, the sites of expression did not. Variations in transgene expression levels are common, and can be affected by the site of integration and by the effect of epigenetic silencing mechanisms on the transgene [18]. Despite the level of variation, the expression of the thylacine *Col2A1* enhancer recapitulated that of the endogenous mouse *Col2a1* gene [15]–[17] and was restricted to the developing chondrocytes. This confirms that the thylacine *Col2a1* gene had a conserved developmental role in cartilage formation, and that its promoter directed expression in chondrocytes in this extinct marsupial mammal. It also suggests that the associated transcription factors and the minimal enhancer element required for inducing expression from this promoter have remained sufficiently conserved between mice and marsupials to direct expression [19], [20] despite approximately 148 million years of divergent evolution between these species [21]–[23].

The minimal 18-bp element [15] required for chondrocyte specific expression is only 17-bp in the thylacine and differs at 4 nucleotides positions compared with the mouse. Furthermore, the sequences of the inverted repeats thought be to essential for the function of this region that flank the minimal element are completely complementary in mouse, rat and human, [15]. However, in the thylacine, the outer repeat is highly conserved but the inner repeat differs by 2 nucleotides and is not complimentary. Despite these differences, the enhancer element was able to function in the developing mouse, suggesting that these differences are not critical for the function of this element. Therefore, the examination of extinct DNA can provide important information about gene function in extant species. Taken together, the results of this study confirm an ancestral role for the thylacine *Col2A1* gene in cartilage formation and demonstrate that it is possible to examine non-coding DNA function from extinct animals *in vivo* in the mouse.

These observations advance previous studies that have so for only examined extinct protein function and only in *in vitro* systems [1], [7]. However, both systems (*in vitro* cell cultures and *in vivo* whole organism models) rely on the availability and compatibility of cofactors within the host cell or embryo in order to examine gene or regulatory sequence function. It is possible that the variations in receptor signalling strength seen in the mammoth and Neanderthal *MC1R* gene analyses *in vitro* [1], [7] did not occur or affect gene function in their native organisms. The native ligand in the mammoth and Neanderthal may have varied to the one used in culture, as could the downstream activation strength of the signalling pathway. Similarly it is possible that the thylacine *Col2A1* enhancer had a different pattern of expression in the developing thylacine embryo to that observed in the mouse, due to the different availability of cofactors required for its activation. The precise function of an extinct gene or regulatory element is impossible to determine without examining every part of the pathway in one system. Results from extinct DNA analyses therefore should be cautiously interpreted.

Thus extinct genomes can be examined by the *in vivo* method described here, in conjunction with *in vitro* techniques, to enable functional analyses of the genes and regulatory elements. This could provide important insights into genome evolution by defining functional features unique to each species. We have demonstrated that non-coding genetic information from an extinct species can be resurrected *in vivo* and in doing so, we have restored to life the genetic potential of a fragment of this extinct mammalian genome.

## Materials and Methods

### Tissues, DNA isolation, amplification, and transgene construction and analyses

Tissues were obtained from three 100-year-old thylacine pouch young specimens fixed in ethanol and one dried 100-year-old adult skin from Museum Victoria, Melbourne. Tissue samples were stored in sterile cryo-vials and transported to the University of Texas. DNA was extracted according to the method described by Pääbo [14] with a control extraction performed on an empty vial. All processing was done in a UV-sterilised bio-containment hood to prevent any contaminating DNA from entering the samples. DNA samples were kept separate and used as separate templates in subsequent PCR reactions. Negative controls (blank-DNA extraction, and no template) were included in each PCR reaction to ensure no contamination of samples. Primers were engineered with *Spe*1 (forward primer) and *Xba*1 (reverse primer) restriction sites to allow for multimerisation (forward 5′ NNNACTAGTGCATTGGGAGATTGGCAGCGAT 3′; reverse 5′ NNNTCTAGAGCTACCTCTTTCGGGGAACTG 3′). The resulting 264-bp PCR product was subcloned and sequenced in both directions for at least three clones from each tissue sample. The sequence was compared to that from possible sources of contamination within the lab using genome databases (human, mouse and tammar wallaby) and was shown to have consistent differences with each species clearly indicating it was derived from a unique source. The fragment was also subjected to phylogenetic analysis to confirm its marsupial origin. Phylogentic relationship of the thylacine PCR fragment was compared to the orthologous region from tammar wallaby, human, mouse and rat using the PHYLIP 3.63 program (University of Washington; http://evolution.genetics.washington.edu/phylip/doc/main.html) using maximum-likelihood, maximum parsimony and neighbor-joining analysis with 1000 replicates. The resulting data was viewed with TREE-view 1.6.6 (http://taxonomy.zoology.gla.ac.uk/rod/treeview.html).

Once sequence identity was confirmed, the clone was digested with *Xba*1 and *Spe*1 and incubated with T4 ligase at 16^o^C for 2 hours. After ligation, the reaction was digested with *Xba*1 and *Spe*1 such that only products multimerised head to tail (ablating the restriction sites) would remain intact. Ligation products corresponding in size to 4 mutimerised copies (1056 bp) were purified. The multimer was ligated to a human β-globin basal promoter fused to *lacZ* followed by a polyadenylation signal. Embryos were stained for LacZ activity [17]. The human β-globin basal promoter-*lacZ*-pA reporter construct on its own does not have tissue-specific activity in transgenic mice [24]. For histological analyses, X-gal stained embryos were postfixed in 4% paraformaldehyde, embedded in paraffin, sectioned and counterstained with eosin [17].
